# The interplay between spatiotemporal overlap and morphology as determinants of microstructure suggests no ‘perfect fit’ in a bat-flower network

**DOI:** 10.1038/s41598-023-29965-3

**Published:** 2023-02-15

**Authors:** Ugo Mendes Diniz, Ludmilla Moura de Souza Aguiar

**Affiliations:** 1 Plant-Insect Interactions, School of Life Sciences, Technische Universität München, Freising, Germany; 2 Graduate Program in Ecology, University of Brasília, Brasília, Brazil; 3 Zoology Department, University of Brasília, Brasília, Brazil

**Keywords:** Ecology, Evolution

## Abstract

Plant–pollinator interactions in diverse tropical communities are often predicted by a combination of ecological variables, yet the interaction drivers between flower-visiting bats and plants at the community level are poorly understood. We assembled a network between Neotropical bats and flowering plants to describe its macrostructure and to test the role of neutral and niche variables in predicting microstructure. We found a moderately generalized network with internally nested modules comprising functionally similar plant and bat species. Modules grouped bats and plants with matching degrees of specialization but had considerable overlap in species morphologies and several inter-module interactions. The spatiotemporal overlap between species, closely followed by morphology, and not abundance, were the best predictors of microstructure, with functional groups of bats also interacting more frequently with plants in certain vegetation types (e.g., frugivores within forests) and seasons (e.g., long-snouted nectarivores in the dry season). Therefore, flower-visiting bats appear to have species-specific niche spaces delimited not only by their ability to exploit certain flower types but also by preferred foraging habitats and the timing of resource availability. The prominent role of resource dissimilarity across vegetation types and seasons likely reflects the heterogeneity of Neotropical savannas, and further research in biomes beyond the Cerrado is needed to better understand the complexity of this system.

## Introduction

One of the main questions addressed by community ecology, particularly in the field of mutualistic interactions, is which ecological variables drive interactions between species^1^. Defining how species in a community interact based on observable mechanisms, such as morphological, spatiotemporal, or phylogenetic matching, is key to understanding mutualism evolution and predicting community and ecosystem dynamics^2,3^. Plant–pollinator interactions are a fertile ground to explore the relative role of different ecological variables in assembling interactions in communities. The diverse adaptive pathways of flowering plants towards biotic pollen vectors have led to a myriad of pollination systems with contrasting degrees of ecological specialization^4^.

For example, interactions in temperate communities with lower species diversity and lower competition for pollinators can often be predicted by species abundances^3,5^, while morphological fit or spatiotemporal synchrony between species either complement the role of abundance or are the main drivers of network structure in diverse tropical communities^6–8^. In such communities, interaction networks are commonly permeated by ‘forbidden links’, interactions that are unlikely or impossible to occur due to unmatching species traits^9^. This non-random distribution of links often leads to higher-scale patterns, such as modularity, a widespread characteristic of mutualistic networks in which species interact more frequently within subgroups^10^.

The interplay between network patterns such as modularity, unrealized links, and drivers of pairwise interactions, however, has been explored in a few groups^9^, with special attention to pollination syndromes such as ornithophily^11,12^ and sphingophily^7,13^, where the correlation between floral morphology and mouthpart length immediately suggest trait matching to some extent. Other tropical syndromes where the morphological fit between species is less clear, such as the pollination of large and accessible flowers by nectarivorous bats, lack significant data on how interactions are assembled at the community level.

The study of bat pollination has historically lagged behind in comparison to other syndromes, but the ecological importance of the syndrome as a key segment of tropical ecosystem functioning has been increasingly recognized and reaffirmed as studies on the system multiplied^14,15^. Several recent works have tried to understand how bat-pollination networks are assembled. In Latin America, flower-visiting bats appear to form generalized networks with non-restrictive flowers that allow visitation by a wide variety of animals^16–18^. Large-scale meta-networks in tropical America have shown that relatively generalized networks emerge in several different regions^19^, and that bat traits such as size and skull length may influence species’ importance in networks locally^20^.

Based on current evidence, the chiropterophilous syndrome seems to generally entail a low morphological and ecological specialization in both flowers and bats. Although the information on whether bats and plants interact according to trait matching (e.g., corresponding tube and rostrum/tongue length) is conflicting^21–23^, longer feeding parts seem to aid bats in acquiring nectar more efficiently and visit a wider variety of plants but they do not necessarily lead to the occurrence of deeper-tubed flowers^24^. Therefore, we should expect a weaker effect of morphological specialization in shaping bat-flower networks, yet the drivers of network microstructure, or the distribution of pairwise interactions, remain thus far unknown for this system.

A few assumptions deriving from the recently described integrative hypothesis of specialization (IHS)^20,25,26^ may shed some light on potential driving factors of bat-flower interactions. The IHS suggests that resource dissimilarity, normally associated with evolutionary constraints, leads to the specialization of consumers (e.g., pollinators) within a cluster of similar resources, improving performance within clusters but reducing performance outside them and leading to higher-scale network patterns such as modularity. Such resource dissimilarity can be translated as functional niche, i.e., clusters formed by similar-shaped flowers (e.g., pollination networks) or closely related plants with similar defensive compounds (e.g., as in herbivory networks), but potentially encompass other aspects of species’ natural history, such as spatial distribution and phenology^26–28^.

Particularly in the context of tropical ecosystems, the spatial and temporal clustering of plant species, i.e., landscape heterogeneity and seasonality, respectively, and the overlap with the habitat requirements and seasonal cycles of animals are often important drivers of the structures of mutualistic networks^9,12,29^. There is a good amount of evidence that bats and present seasonal shifts in activity related to the availability floral resources^22,30,31^ and that bat communities undergo species turnover along environmental gradients as bats can be selective in terms of the habitat in which they roost and forage^32,33^. Therefore, as an alternative to morphology, spatiotemporal overlap could serve as a better ecological driver of interactions between bats and plants, especially in seasonal and heterogeneous ecosystems such as seasonal deciduous forests, savannas, and dry forests.

Therefore, given the apparent small role of morphological fit within bat-pollination, the generalized networks that have been described for this system, and the assumptions deriving from the IHS, we assembled a comprehensive pollen transfer network between flowering plants and phyllostomid bats in a highly seasonal and heterogeneous Neotropical savanna to address its and driving mechanisms, while predicting a (i) generalized, non-modular network with a weak or negligible role of species-level morphological specialization as a driver of pairwise interactions. Alternatively, interactions would be driven purely by (ii) species abundances due to the prolific behavior of bats and low restriction of bats and plant species, by the (iii) spatiotemporal overlap between species if a strong spatial (landscape heterogeneity) and temporal (seasonality) components are present in the community, or by (iv) a synergistic combination of the spatiotemporal clustering of resources with other variables.

## Materials and methods

### Study site

The study was conducted in the Brasília National Park (PNB), Federal District, Brazil (15º39′57″ S; 47º59′38″ W), a 42.355 ha Protected Area with a typical vegetation configuration found in the Cerrado of the central highlands of Brazil, i.e., a mosaic of gallery forest patches along rivers surrounded by a matrix of savannas and grasslands^34^. The climate in the region falls into the Aw category in the Köppen scale, categorizing a tropical wet savanna, with marked rainy (October to March) and dry (April to September) seasons.

We carried out the study in eight fixed sampling sites scattered evenly throughout the PNB and separated by at least two kilometers from one another (Supplementary Fig. S1). The sites consisted of four cerrado sensu stricto sites (bushy savanna containing low stature trees); two gallery forest edges sites (ca. 5 m from forest edges, containing a transitional community), and two gallery forest interior sites. These three types reflect the overall availability of habitat types in the reserve (excluding grasslands) and are the most appropriate foraging areas to sample interactions as bat-visited plants are either bushes, trees, or epiphytes, but rarely herbs^35^.

### Bat and interaction samplings

We sampled bat-plant interactions using pollen loads collected from bat individuals captured in the course of one phenological year, thus configuring an animal-centered sampling. We carried out monthly field campaigns to capture bats from October 2019 to February 2020, from August to September 2020, and from March to July 2021. In each month, we carried out eight sampling nights during periods of low moonlight intensity, each associated with one of the eight sites. Each night, we set 10 mist nets (2.6 × 12 m, polyester, denier 75/2, 36 mm mesh size, Avinet NET-PTX, Japan) at ground level randomly within the site, which were opened at sunset and closed after six hours. We accumulated a total sampling effort of 552 net-hours, 28,704 m^2^ of net area, or 172,224 m^2^h sensu Straube and Bianconi^36^.

All captured bats were sampled for pollen, irrespective of family or feeding guild. We used glycerinated and stained gelatin cubes to collect pollen grains from the external body of bats (head, torso, wings, and uropatagium). Samples were stored individually, and care was taken not to cross-contaminate samples. Pollen types were identified by light microscopy, and palynomorphs were identified to the lowest-possible taxonomical level using an extensive personal reference pollen collection from plants from the PNB (details in next section). Palynomorphs were sometimes classified to the genus or family level or grouped in entities representing more than one species. Any palynomorph numbering five or fewer grains in one sample was considered contamination, alongside any anemophilous species irrespective of pollen number.

Bats were identified using a specialized key^37^ and four ecomorphological variables were measured for each individual. (i) Forearm length and (ii) body mass were used to calculate the body condition index (BCI), a proxy of body robustness^38^, where higher BCI values indicate larger and heavier bats, which are less effective in interacting with flowers in general due to a lack of hovering behavior, the incapability of interacting with delicate flowers that cannot sustain them, a lower maneuverability and higher energetic requirements^39^. Moreover, we measured (iii) longest skull length (distance from the edge of the occipital region to the anterior edge of the lower lip) and (iv) rostrum length (distance from the anterior edge of the eye to the anterior edge of the lower lip) to calculate the rostrum-skull ratio (RSR), a proxy of morphological specialization to nectar consumption^23^. Higher RSR values indicate bats with proportionally longer rostra in relation to total skull length. Longer rostra in bats are associated with a weaker bite force and thus less effective in consuming harder food items such as fruits and insects, thus suggesting a higher adaptation to towards nectar^40,41^. Bats were then tagged with aluminum bands for individualization and released afterward. To evaluate the sampling completeness of the bat community and of the pollen types found on bats, we employed the Chao1 asymptotic species richness estimator and an individual-based sampling effort to estimate and plot rarefaction curves, calculating sampling completeness according to Chacoff et al.^42^.

All methods were carried out in accordance with relevant guidelines and regulations. The permits to capture, handle and collect bats were granted by the Ethical Council for the Usage of Animals (CEUA) of the University of Brasília (permit 23106.119660/2019-07) and the Instituto Chico Mendes de Conservação da Biodiversidade (ICMBio) (permit: SISBIO 70268). Vouchers of each species, when the collection was possible, were deposited in the Mammal Collection of the University of Brasília.

### Assessment of the plant community

In each of the eight sampling sites, we delimited a 1000 × 10 m transect, each of which was walked monthly for one phenological year (January and February 2020, August to December 2020, and March to July 2021) to build a floristic inventory of plants of interest and to estimate their monthly abundance of flowering individuals. Plant species of interest were any potential partner for bats, which included species already known to be pollinated by bats, presenting chiropterophilous traits sensu Faegri and Van Der Pijl^43^, or any plant that could be accessed by and reward bats, whose flowers passes all the three following criteria:

(i) Nectar or pollen is presented as the primary reward to visitors. (ii) Corolla diameter of 1 cm or more. This criterion excludes small generalist and insect-pollinated flowers where the visitation by bats is mechanically unlikely. It applies to the corolla diameter in non-tubular flowers or the diameter of the tube opening. Exceptions were small and actinomorphic flowers aggregated in one larger pollination unit (pseudanthia) where the 1 cm threshold was applied to inflorescence diameter. (iii) Reward must be promptly available for bats. This criterion excludes species with selective morphological mechanisms, such as quill-shaped bee-pollinated flowers or flowers with long and narrow calcars.

All flowering individuals of interest species found in the transects were registered. A variable number of flowers/inflorescences (n = 5–18) were collected per species for morphometric analysis. For each species, we calculated floral tube length (FTL), corresponding to the distance between the base of the corolla, calyx, or hypanthium (depending on the species) to its opening, and the corolla’s outermost diameter (COD), which corresponds to the diameter of the corolla opening (tubular flowers) or simply the corolla diameter (non-tubular flowers). For pseudanthia-forming species, inflorescence width was measured. Pseudanthia and non-tubular flowers received a dummy FTL value of 0.1 mm to represent low restriction and enable later calculations. Finally, we collected reference pollen samples from all species from anthers of open flowers, which were used to identify pollen types found on bats. For plant species found in pollen loads but not in the PNB, measures were taken from plants found either on the outskirts of the site (*Inga* spp.) or from dried material in an online database (*Ceiba pentandra,* in https://specieslink.net/) using the ImageJ software^44^. Vouchers were deposited in the Herbarium of the Botany Department, University of Brasília.

### Data analysis

#### Network macrostructure

We built a weighted adjacency matrix *i* x *j*, where cells corresponded to the number of individuals of bat species *i* that interacted with plant species or morphotype *j*. All edges corresponding to legitimate interactions were included. With this matrix, we calculated three structural metrics to describe the network’s macrostructure. First, weighted modularity (Qw), calculated by the DIRTLPAwb + algorithm^45^. A modular network comprises subgroups of species in which interactions are stronger and more frequent than species out of these subgroups^10^, which may reveal functional groups in the network^9^. Qw varies from zero to one, the latter representing a perfectly modular network.

Second, complementary specialization through the H_2_′ metric^46^. It quantifies how unique, on average, are the interactions made by species in the network, considering interaction weights and correcting for network size. It varies from zero to one, the latter corresponding to a specialized network where interactions perfectly complement each other because species do not share partners.

Lastly, nestedness, using the weighted WNODA metric^25^. Nested networks are characterized by interaction asymmetries, where peripheral species are only a subset of the pool of species with which generalists interact^47^. The index was normalized to vary from zero to one, with one representing a perfectly nested network. Given that the network has a modular structure, we also tested for a compound topology, i.e., the existence of distinct network patterns within network modules, by calculating intra-module WNODA and between-module WNODA^36^. Internally nested modules appear in networks in which consumers specialize in groups of dissimilar or clustered resources and suggest the existence of distinct functional groups of consumers^25,48^. Metric significance (Qw, H_2_′, and WNODA) was assessed using a Monte Carlo procedure based on a null model. We used the *vaznull* model^3^, where random matrices are created by preserving the connectance of the observed matrix but allowing marginal totals to vary. One thousand matrices were generated and metrics were calculated for each of them. Metric significance (*p*) corresponded to the number of times the null model delivered a value equal to or higher than the observed metric, divided by the number of matrices. The significance threshold was considered *p* ≤ 0.05.

Given a modular structure, we followed the framework of Phillips et al.^49^ that correlates network concepts (especially modularity) with the distribution of morphological variables of pollinators to unveil patterns of niche divergence in pollination networks. Given the most parsimonious module configuration suggested by the algorithm, we compared modules in terms of the distribution of morphological variables of the bat (RCR and BCI) and plant (FTL and COD) species that composed the module. Differences between modules means were tested with one-way ANOVAs.

#### Drivers of network microstructure

The role of different ecological variables in determining pairwise interaction frequencies was assessed using a probability matrices approach^3^. This framework considers that an interaction matrix *Y* is a product of several probability matrices of the same size as *Y*, with each matrix representing the probability of species interacting based on an ecological mechanism. Thus, adapting it to our objectives, we have Eq. (1):

1 $$\mathrm{Y}=\mathrm{f}(\mathrm{A},\mathrm{ M },\mathrm{P},\mathrm{ S})$$

where Y is the observed interaction matrix, and a function of interaction probability matrices based on species relative abundances (A), representing neutrality as species interact by chance; species morphological specialization (M), phenological overlap (P), and spatial overlap (S). We built models containing each of these matrices in the following ways:

Relative abundance (A): matrix cells were the products of the relative abundances of bat and plant species. The relative abundances of bats were determined through capture frequencies (each species’ capture frequency divided by all captures, excluding recaptures) and the relative abundances of plants were determined by the number of flowering individuals recorded in transections (each species’ summed abundance in all transects and all months divided by the pooled abundance of all species in the network). Cell values were normalized to sum one.

Morphological specialization (M): cells were the probability of species interacting based on their matching degree of morphological specialization. Morphologically specialized bats (i.e., longer rostra and smaller size) are more likely to interact with morphologically specialized flowers (i.e., longer tubes and narrower corollas), while unspecialized bats are more likely to interact with unspecialized, accessible flowers. For this purpose, we calculated a bat specialization index (BSI) as the ratio between RCR and BCI, where higher BSI values indicate overall lower body robustness and longer snout length. Likewise, the flower specialization index (FSI) was calculated for plants as the ratio between FTL and COD, where higher values indicate smaller, narrower, long-tubed flowers that require specialized morphology and behavior from bats for visitation. BSI and FTL were normalized to range between zero and one and were averaged between individuals of each species of bat or plant. Therefore, interaction probabilities were calculated as in Eq. (2):

2 $${P}_{i,j}=1-|{BSI}_{i}-{FSI}_{j}|$$

where *P*_i,j_ is the interaction probability between bat species *i* and plant species *j* and |*BSI*_*i*_ – *FSI*_*j*_| is the absolute difference between bat and plant specialization indexes. Similar index values (two morphologically specialized or unspecialized species interacting) lead to a low difference in specialization and thus to a high probability of interaction (*P*_i,j_ → 1), whereas the interaction between a morphologically specialized and a morphologically unspecialized species leads to a high absolute difference and thus lower probability of interaction (*P*_i,j_ → 0). Cell values of the resulting matrix were normalized to sum one.

Phenological overlap (P): cells were the probability of species interacting based on temporal synchrony, calculated as the number of months that individuals of bat species *i* and flowering individuals of plant species *j* co-occurred in the research site, pooling all capture sites/transections. Cell values were normalized to sum one.

Spatial overlap (S): cells were the probability of species interacting based on their co-occurrence over small-scale distances and vegetation types, calculated as the number of individuals from a bat species *i* captured in sampling sites where the plant species *j* was registered in the transection, considering all capture months. Cell values were normalized to sum one.

Because more than one ecological mechanism may simultaneously drive interactions^3,9^, we built an additional set of seven models resultant from the element-wise multiplication of individual probability matrices:

- SP: The spatial and temporal distribution of species work simultaneously in driving a resource turnover in the community, driving interactions.
- AS: Abundance drives interactions between bats and plants, but within spatially clustered resources in the landscape caused by a turnover in species distributions.
- AP: Abundance drives interactions between bats and plants, but within temporally clustered resources caused by a seasonal distribution of resources.
- APS: Abundance drives interactions between bats and plants, but within resource clusters that emerge by a simultaneous temporal and spatial aggregation.
- MS: Similar to AS, but morphology drives interactions within spatial clusters.
- MP: Similar to MP, but morphology drives interactions within temporal clusters.
- MPS: Similar to APS, but morphology drives interactions within spatiotemporal clusters.

Finally, we created a benchmark null model in which all cells in the matrix had the same probability value. All the compound matrices and the null model were also normalized to sum one.

To compare the fit of these probability models with the real data, we conducted a maximum likelihood analysis^3,9^. We calculated the likelihood of each of these models in predicting the observed interaction matrix, assuming a multinomial distribution for the probability of interaction between species^12^. To compare model fit, we calculated the Akaike Information Criterion (AIC) for each model and their variation in AIC (ΔAIC) in relation to the best-fitting model. The number of species used in the probability matrices was considered the number of model parameters to penalize model complexity. Intending to assess whether nectarivorous bats and non-nectarivorous bats assembly sub-networks with different assembly rules, we created two partial networks from the observed matrix. One contained nectarivores only (subfamilies Glossophaginae and Lonchophyllinae) and their interactions, and the other contained frugivore and insectivore bats and their interactions. We repeated the likelihood procedure for these two partial networks.

To conduct the likelihood analysis, we excluded plant species from the network that could not have their interaction probabilities measured, such as species found in pollen samples but not registered in the park or pollen types that could not be identified to the species level. Therefore, the interaction network *Y* and probability matrices did not include these species (details in Supplementary Table S1).

#### Software

Analyses were performed in R 3.6.0^50^. Network metrics and null models were generated with the *bipartite* package^51^, and the sampling completeness analysis was performed with the *vegan* package^52^. Gephi 0.9.2^53^ was used to draw the graph.

## Results

### Bat community and spatiotemporal trends

We captured a total of 386 bats from 23 species and three families (Supplementary Table 2). From this pool, 162 bats from 13 species belonging to Phyllostomidae and subfamilies Carollinae, Glossophaginae, Lonchophyllinae, Micronycterinae, and Stenodermatinae were floral visitors, thus including nectarivores, frugivores, and one insectivore species. The asymptotic species richness estimator revealed high sampling completeness for Phyllostomidae, the only family acting as floral visitors (mean 93.3%, or 14 out of 15 ± 2.29 SE) (Supplementary Fig. S2).

**Figure 1:**
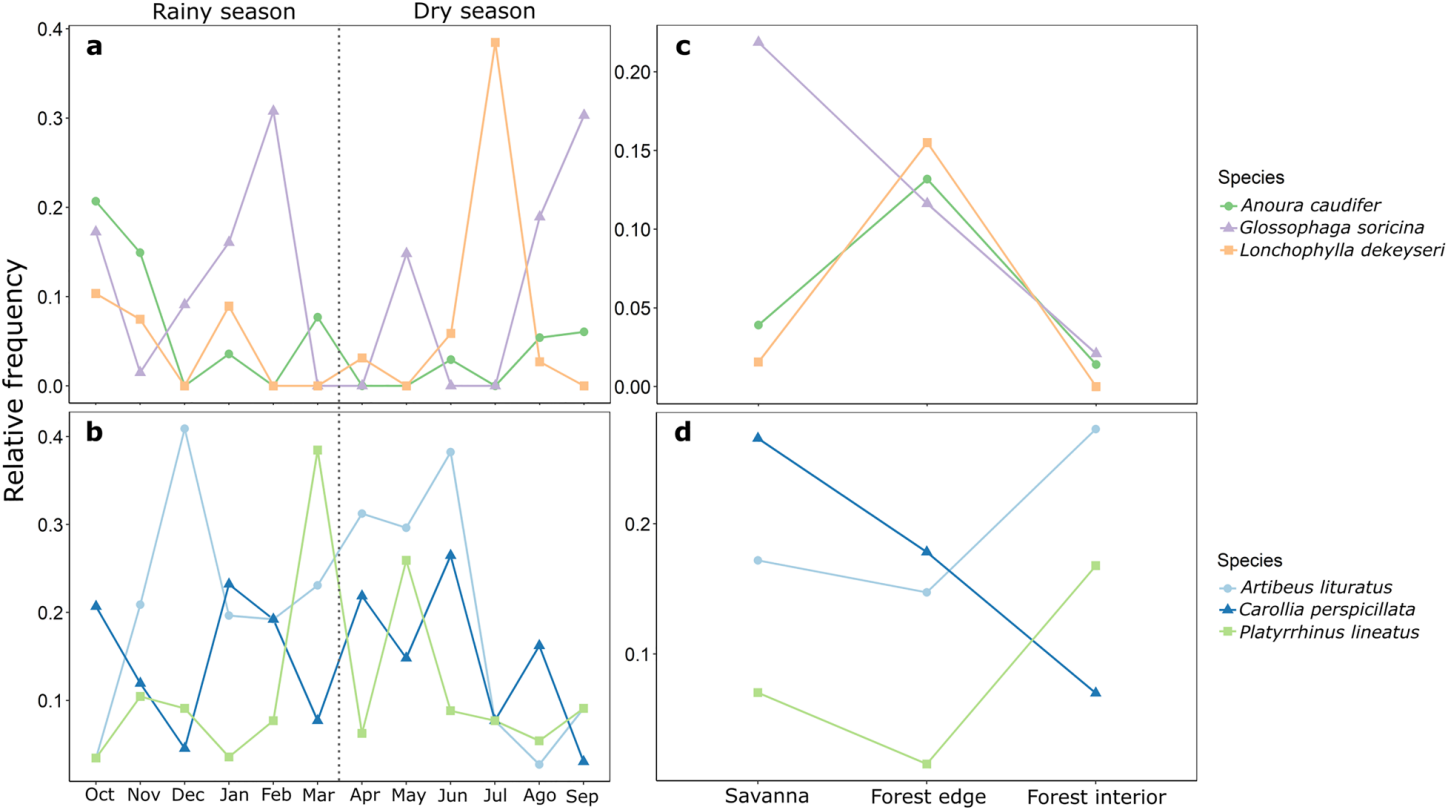
Spatiotemporal trends of bat capture rates of the most frequent floral visitors in the study site. Temporal trends in the relative frequency (in relation to all captured in a given month) of specialized nectarivorous bats (**a**) and mainly frugivorous bats (**b**) throughout the year. A dashed line separates the dry and rainy seasons. Spatial trends in the relative frequency of nectarivorous (**c**) and frugivorous bats (**d**) according to habitat type.

The most frequent floral visitors in the community (Supplementary Table S2) showed distinguishable species-specific spatial and temporal patterns in activity. The nectarivores *Glossophaga soricina* and *Anoura caudifer* (Glossophaginae) were much more common during the transition between dry and wet seasons and during the peak wet season but were replaced by *Lonchophylla dekeyseri* (Lonchophyllinae) at peak dry season (Fig. 1a). Frugivores were temporally variable, without a clear trend (Fig. 1b). In terms of spatial trends, *G. soricina* dominated savanna sites, where other nectarivores were rarely recorded (Fig. 1c) but decreased steeply towards forests. *Anoura caudifer* and *L. dekeyseri* were more abundant in forest edges, and all nectarivores were rare or unrecorded in forests. Frugivores were again variable but were overall more common in forest interiors except for *Carollia perspicillata*, which increased in abundance towards savannas (Fig. 1d).

### Interactions with plants

We recorded 302 interactions with 35 different pollen morphotypes from 16 plant families, from which 18 were identified at the species level, two to the species group level (*Bauhinia* groups, containing species with similar flower and pollen morphologies), and four to the genus level (Supplementary Table S1). The remaining were identified either as broader taxonomical types or at the family level. Two types remained unidentified. The species included both chiropterophilous and non-chiropterophilous species. The sampling of chiropterophilous plants based on pollen loads was complete (15 out of 15 ± 0) (Supplementary Fig. S2). Therefore, we consider that the main interactions in the network were sufficiently sampled. When including the pollen types of non-chiropterophilous plants in the analysis, a mean of 50% (35 out of 70 ± 25.59) of the estimated community was sampled, resulting from singletons found occasionally in samples.

### Network macrostructure

**Figure 2:**
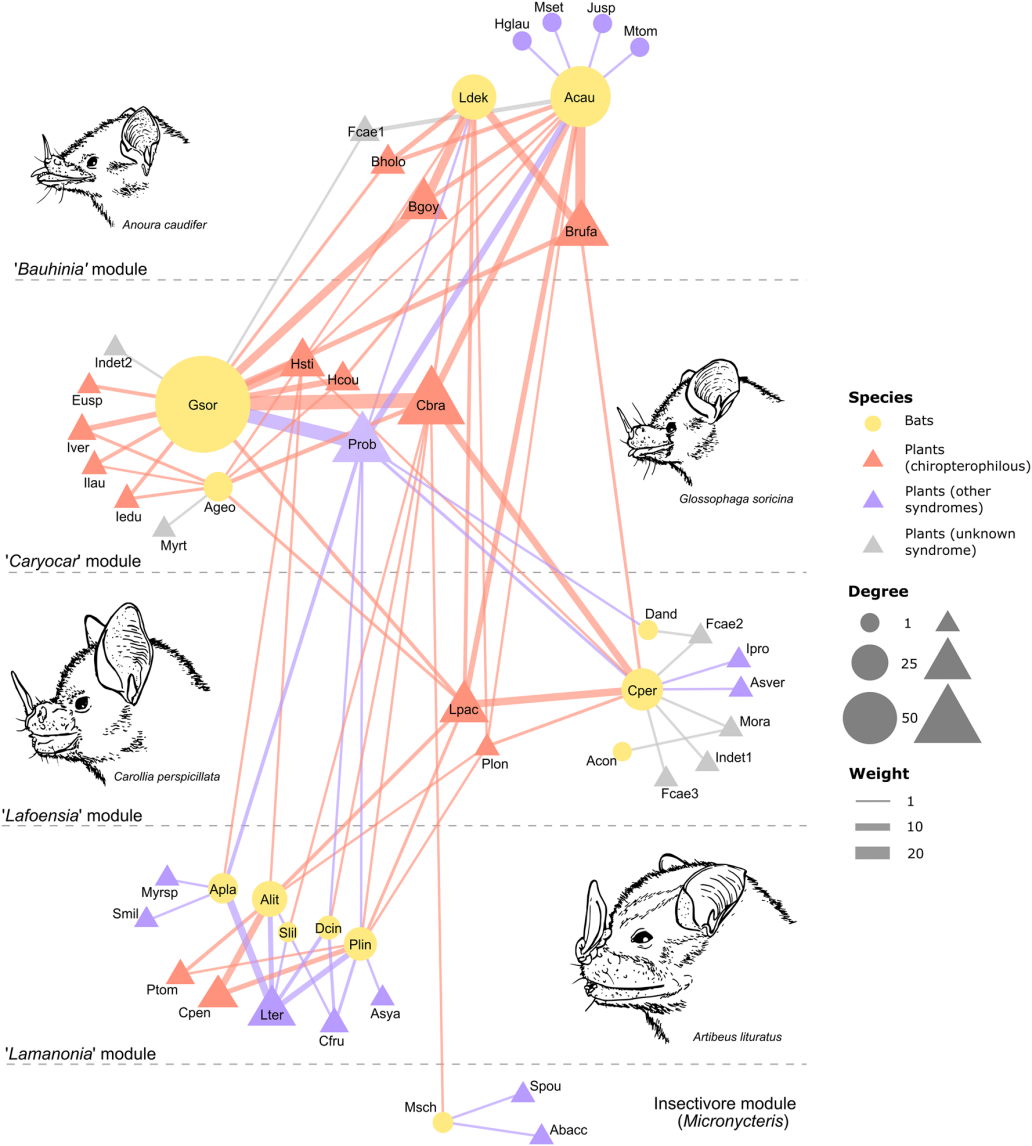
Interaction network between flower-visiting phyllostomid bats and plants in a savanna of central Brazil. Nodes represent species and lines, pairwise interactions. Line width corresponds to interaction weight (frequency) and node size to a species’ degree, or the sum of a species’ interactions. Plants are divided into chiropterophilous, non-chiropterophilous, or unknown syndromes. Modules in the network are divided by dashed lines and accompanied by a schematic illustration of the most important bat species in the module. Species codes are found in Supplementary Tables S1 and S2.

**Figure 3:**
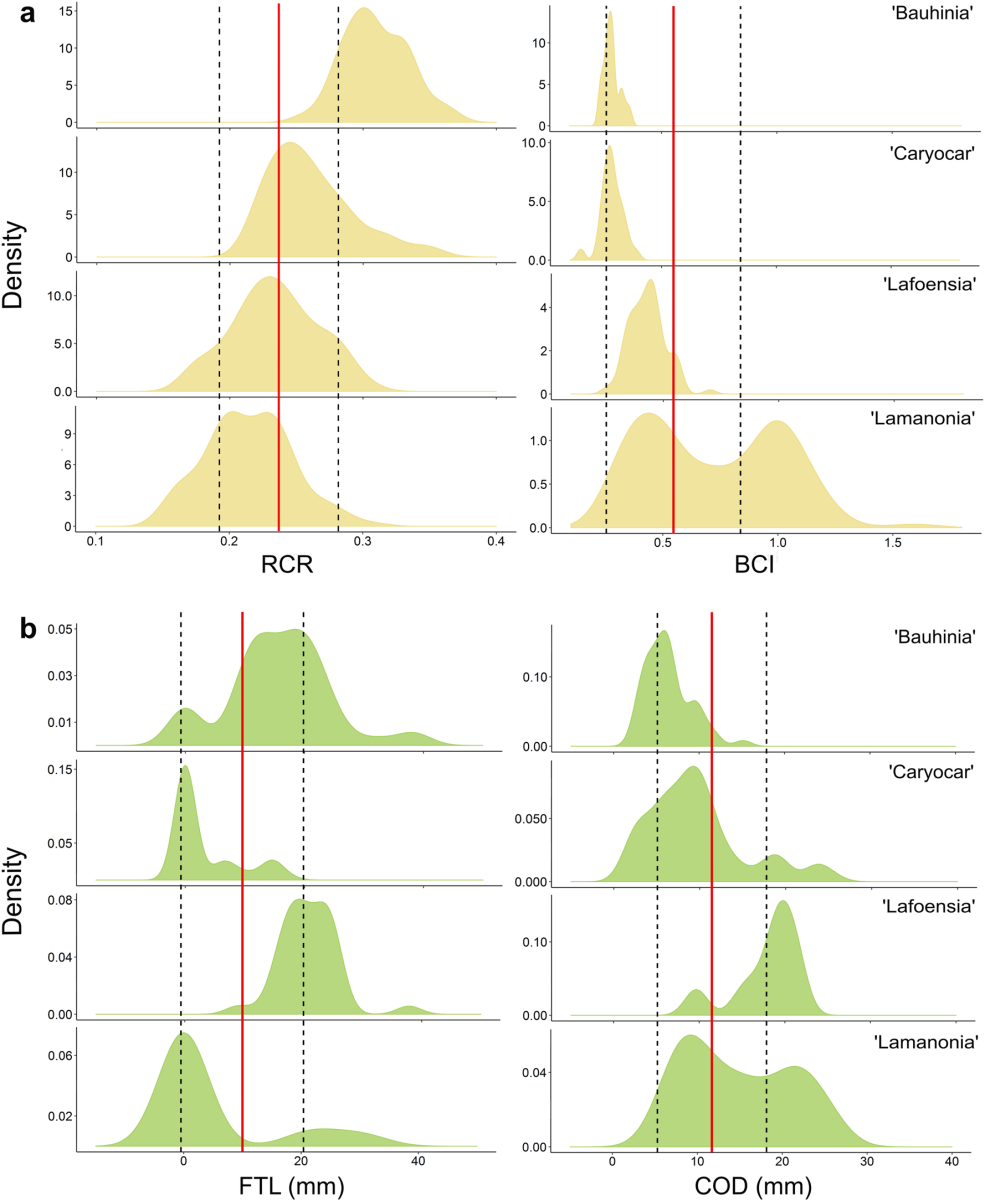
Density distribution of morphological variables from species in the network across modules. (**a**) Bats (RCR—rostrum-cranium ratio, and BCI—body condition index); (**b**) plants (FTL—floral tube length, and COD corolla outermost diameter). Module names correspond to those in Fig. 1. Solid red lines show the mean of each variable for all species pooled, and dashed lines indicate the standard deviation.

The interaction network was moderately modular (Qw = 0.42, p = 0.00) and generalized (H’_2_ = 0.37, p = 0.00). It showed little nestedness (WNODA = 22.20, p = 0.19), but a compound topology emerged with a much higher within-module nestedness (WNODA = 54.46) than between-module nestedness (WNODA = 13.89). Module configuration was correlated with bat feeding guilds (Figs. 2, 3). Small and long-snouted nectar bats (*L. dekeyseri* and *A. caudifer*) were grouped mostly with tube-flowered species of the genus *Bauhinia* spp. (‘Bauhinia’ module). The larger *A. geoffroyi* and the short-snouted *G. soricina* were placed in the same module with mostly open-flowered or short-tubed species such as *Caryocar brasiliense* and *Psittacanthus robustus*, respectively (*‘*Caryocar’ module). Two modules were formed solely by frugivores and the last by the insectivorous *Micronycteris schimdtorum*, the latter with only three interactions in total. The frugivore modules were either dominated by the savanna-specialist *Carollia perspicillata* interacting with robust chiropterophilous flowers with wide corollas (e.g., *Lafoensia pacari, Ceiba pentandra*, *Pseudobombax* spp.) (‘Lafoensia’ module), or by forest-specialists *Artibeus* spp. interacting with non-chiropterophilous, pseudanthia-forming species (e.g., *Lamanonia ternata* and *Combretum fruticosum*) (‘Lamanonia’ module)*.*

All morphological variables varied significantly across modules. Rostrum-cranium ratios were significantly higher in the ‘Bauhinia’ module and successively lower (F_3,360_ = 95.52, p < 0.0001, all pairwise comparisons with p < 0.001) (Fig. 3a). Body condition index was similar in both nectarivore modules (p = 0.99), but much lower than in other modules (F_3,358_ = 89.72, p < 0.0001, all other pairwise comparisons with p < 0.05) (Fig. 3a). There was also variation in floral tube length (F_3,204_ = 57.68, p < 0.0001) and diameter of the corolla/floral tube opening (F_3,202_ = 48.17, p < 0.0001) across modules (Fig. 3b), but with a less clear pattern. The ‘Caryocar’ module had mostly open-flowered species, alongside the ‘Lamanonia’ module (p = 0.58, but p < 0.05 for all other pairwise comparisons), contrasting with the longer-tubed flowers in the ‘Bauhinia’ and ‘Lafoensia’ modules. However, these latter two contrasted sharply in terms of corolla opening, with long-tubed *Bauhinia* spp. being much more restrictive than the large and wide-tubed flowers in the ‘Lafoensia’ module. The ‘Lamanonia’ module included large open flowers or pseudanthia. All modules differed in corolla diameter (p < 0.005 for all pairwise comparisons).

### Drivers of microstructure

**Figure 4:**
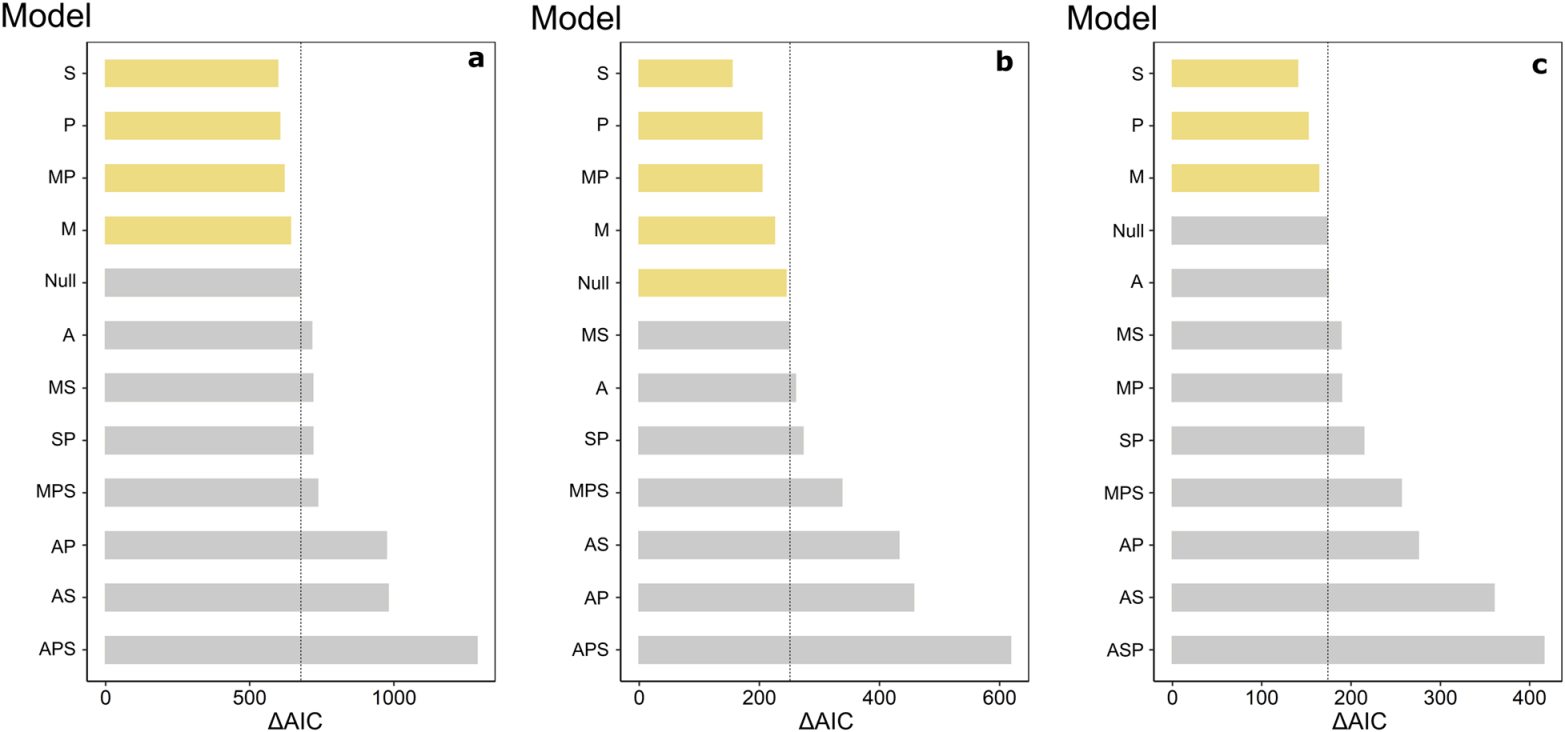
The likelihood of different interaction probability models of explaining pairwise interaction frequencies in the observed network. Model fit is expressed in their variation in the Akaike Information Criterion relative to the observed matrix. Models are organized from best fit (top) to worst fit (bottom). (**a**) Full network, (**b**) nectarivores only, and (**c**) other guilds only. Model labels—M: morphological specialization, A: relative abundance, S: spatial overlap, P: phenological overlap, Null: benchmark null model.

Out of the 11 probability models, spatial overlap and temporal overlap alone were the two best predictors of pairwise interaction frequencies across all networks (full, nectarivore only, and other guilds only), followed closely by morphology or by a combination of morphology and temporal overlap (Fig. 4). Spatial overlap was the best-fitting model in all three cases. Abundance performed consistently worse than the null model, as well of all models containing this variable, and most compound models (Fig. 4).

## Discussion

We described a pollen transport network by bats in a South American savanna, bringing a novel empirical observation of a Neotropical bat-flower network after a few previous studies^16–18^ and shedding light on the drivers of network microstructure. Although fewer bats were captured in comparison to other mist-netting works in the Cerrado (which commonly vary between ca. 400–800 captures), our mist-netting effort was considerably higher and total species diversity was comparable or higher^54–56^. Fewer captures may be due to a stochastic component, lower local abundance or to the random placement of nets in the sparse cerrado vegetation, but the total accumulated mist-netting effort, coupled to a good taxonomic resolution and high sampling completeness of bats (Fig. S2) may warrant a reasonable interpretation of network metrics^57^. In terms of macrostructure, the network conformed with previous evidence of a generalized system comprising mostly plants with low morphological restriction and bats capable of exploiting a wide variety of resource plants, resulting in moderate modularity. Additionally, we showed that the frequency of pairwise interactions is driven by a set of ecological variables.

Despite being a good example of ecological generalists^17^, flower-visiting bats and their resource plants did not form a neutrally assembled network, in which interactions would emerge solely based on the probability of species interacting according to their relative abundances, which appears as a strong determinant of microstructure among several pollination networks^58^. Instead, both the patterns behind module separation (network macrostructure) and non-random distribution of pairwise interaction frequencies (microstructure) indicate an effect of morphological traits of bats and plants on network assembly.

Module separation was constrained by species phylogeny and associated with the functional morphology of both bat groups and their visited plants, which serves as a smaller-scale parallel to the role of phylogenetic constraints as the first filter of interactions between bat-plant interactions at large, continental scales^20^. This morphological “filter” seems to operate via the typical forbidden links mechanism, excluding interactions that are mechanically unlikely to occur (e.g., short-snouted and non-hovering frugivores and delicate tubular flowers) and leading to higher-scale network patterns. Although a single-taxon network in a broader sense (i.e., comprising the family Phyllostomidae only), the inclusion of frugivores of the Stenodermatinae and Carollinae subfamilies likely was the cause of the emergence of the observed compound structure, as these groups can show a good performance as consumers on a specific type of exploitable floral resource (robust and accessible flowers or inflorescences), whereas bats with morphologies efficient for nectar extraction may specialize in another subset of more restrictive flowers (e.g., *Bauhinia* spp.).

Differences in bat phylogenetic history and dissimilarities in plant morphology thus likely accounted greatly for the observed modules in the compound-topology network, as normally observed highly specialized antagonistic and/or multi-taxa networks^20,26,59^. Additionally, the observed module separation highlights that snout length and body condition in bats can be seen as proxies for morphological specialization toward floral resources, as suggested by several authors^30,60,61^ while also translating into community-wide patterns of interaction by playing a role in resource partitioning. Nonetheless, the somewhat relaxed distribution of interactions and some overlap in morphological variables of bats and plants between modules suggest that bats are not tightly restricted by plant morphology, and the absence of a few possible interactions (e.g., between nectarivores and several chiropterophilous and non-chiropterophilous plants in the frugivore modules) suggest that other variables beyond morphology are driving interaction in the community. Rather, results show that the flower-visitor niche of bats in the region is delimited, in fact, mostly by time and space.

The temporal and spatial overlap between species were the best predictors of pairwise interactions, followed closely by morphology. Different bats and plants showed higher abundances in distinct vegetation types or periods of the year, leading to higher interaction frequencies based on co-occurrence (e.g., Stenodermatinae frugivores and forest plants) and temporal synchrony (e.g., *L. dekeyseri* and *Bauhinia* spp. during the dry season). This corroborates with the IHS, which suggests that dissimilar or clustered resources lead to consumer specialization within clusters in order to maximize consumer gain^26,48^. The heterogeneous nature of the South American savannas, where plant species are often restricted to vegetation types in the grassland-forest gradient and have seasonal, complementary flowering periods^34,62^, likely had a strong effect on the assemblage of the network by leading to resource partitioning among bats. Although it may not hold for environments with a more homogeneous vegetation structure or a less stark temporal contrasts in resource availability, the spatiotemporal differences in resource availability could be considered the most important type of resource dissimilarity in the studied community, and likely worked synergistically alongside morphological constraints and bat phylogenetic history to drive network structure.

A closer look into the Stenodermatinae frugivore bats in the network provides a good example of the spatial and temporal components in effect. These bats are generally an overlooked component of bat-pollination interactions despite consuming nectar and pollen during times of fruit shortage. Such times correspond to the dry season in the Cerrado^63^, and especially during this season, opportunistic frugivores emerged as an important component of the pollen-transport network. *Ceiba pentandra*, for example, has large and resource-dense inflorescences that bloom in the dry season^64^ and was exclusively visited by large frugivores. Additionally, these bats roost in trees within forest patches, where they also tend to forage due to the higher availability of chiropterocoric plants in comparison to the matrix^32^. Finally, the potential resources for these bats within this setting were a product of the primary morphological filter, as flowers need to be large and allow clinging visits^65^, like the pseudanthia of *L. ternata* and *Combretum fruticosum* or the large inflorescences of *C. pentandra*. The dominance of frugivores as floral visitors in forest sites probably excluded nectarivores from the forest through competitive pressure since bat species that are usually found in forests, such as *A. caudifer*^66^, were not registered in these environments. Another possible instance of niche partitioning was seen in the frugivore *Carollia perspicillata* (Carolliinae), which avoided forests and specialized in the savanna/edge plant *Lafoensia pacari* but could also visit plants such as the tubed *Bauhinia rufa* due to its intermediate snout length and body condition.

Likewise, the interaction patterns among specialized nectar bats also suggest a combination of interaction drivers. Nectar bats tend to consume hard food items (e.g., insects and fruits) in periods of scarcity of floral resources^67^, but bats with longer rostra and weaker bites are less prone to do so^40^. At the dry-rainy season transition, the common and short-snouted *G. soricina* peaked in activity while feeding on the savanna species *Caryocar brasiliense* and *Hymenaea stigonocarpa*, whose short but intense flowering periods offer large amounts of accessible nectar^68^ and are more profitable for short-snouted bats^23^. At peak dry season, *G. soricina* was not recorded in the site and only the long-snouted, small, and more specialized *L. dekeyseri* and *A. caudifer* were detected, dominating the floral-visitor niche in forest edge sites and feeding mainly on *Bauhinia* spp., which offer smaller flowers and less abundant, enclosed nectar^69^.

It is thus clear that the energetical constraints of bats interact with functional morphology to determine foraging decisions. In seasonal ecosystems, nectar bats with longer rostra can effectively consume smaller quantities of nectar and sustain themselves longer as floral visitors by exploring a wider variety of plants^24^, while less morphologically specialized bats shift to other food items^22^. In the Cerrado, the dry season is deemed a period of higher nectar availability for bats, while the rainy season poses flower shortage^31^. However, based on our results, the dry season seems to be crucial for the most specialized nectar bats in the region, which can consume and process smaller nectar volumes more effectively, thus partitioning their niche with other nectarivores, as previously reported in Central America^23^.

Therefore, rather than a tight key-lock relationship, the degree of morphological specialization in bats appears to determine their capacity to sustain themselves in the community as nectar consumers before seeking other types of resources. Our models did not consider variables related to foraging optimization, and still left a considerable amount of variation in the original matrix unexplained. The best-fitting model, for instance (*P* for the nectarivore-only network) (ΔAIC_p_ = 90.02) was not considerably far ahead in comparison to the mull model in terms of fit (ΔAIC_null_ = 154.21) (Fig. 4), and perhaps the inclusion of other variables related to foraging optimization such as nectar volume, concentration, and energy density of chiropterophilous plants, as well as bat energetical requirements and basal metabolism, could be introduced in further studies to help explain interaction assemblage.

## Conclusions

The interactions between flower-visiting bats and plants were not randomly assembled and were a result of different, interacting drivers. The niche of flower-visiting bats in the region seems to be delimited to some extent by a spatial, temporal, and morphological component, and their capacity to exploit different floral types and fine-tune their foraging strategy based on the unequal distribution of resources in time and space leads to niche partitioning. Bat phylogenetic history also plays a role in network macrostructure as frugivorous bats tended to visit a different subset of plants, highlighting the importance of considering a diverse and inclusive assemblage of bat pollinators. The effect of the temporal and spatial components could reflect the highly heterogeneous and seasonal savannas, and further works may reveal different assembly patterns along large biogeographical gradients. Likewise, the inclusion of more variables, such as those related to resource and energy availability, may help in understanding the assembly rules of this system.

We also highlight that empirical works on bat-flower networks in the Neotropics thus far had an animal-centered approach, which is generally more comprehensive than a phytocentric approach but may lead to interactions with plants outside of the research area due to pollinator home ranges^70^. In the present study, a key limitation was the exclusion of several plant species from the microstructure analysis due to the lack of spatiotemporal data, which may be overcome by combining different sampling strategies, which yield more comprehensive results^71^.

## Supplementary Information

Supplementary Information.

## Data Availability

All data produced from this study are provided in this manuscript. The datasets and R script used to conduct the analysis in the current study are available in a public GitHub repository (10.5281/zenodo.7567633).
