# Supplementary material for: The interplay between spatiotemporal overlap and morphology as determinants of microstructure suggests no ‘perfect fit’ in a bat-flower network
Source: Sci Rep. 2023 Feb 15;13:2737. doi: 10.1038/s41598-023-29965-3 (PMC9932087; doi:10.1038/s41598-023-29965-3)
Supplement: Supplementary file 1 — Supplementary Information. [file 41598_2023_29965_MOESM1_ESM.docx]

Supplementary Information

**The interplay between spatiotemporal overlap and morphology as determinants of microstructure suggests no ‘perfect fit’ in a bat-flower network**

Ugo Mendes Diniz^1,2 *^, Ludmilla Moura de Souza Aguiar^2,3^

^1^Research Department Life Science Systems, Technische Universität München, Freising, Germany.

^2^Graduate Program in Ecology, University of Brasília, Brasília, Brazil.

^3^Zoology department, University of Brasília, Brazil

*Corresponding author

E-mail address: ugo.diniz@tum.de

Tel.: +49 0152 02746484

^
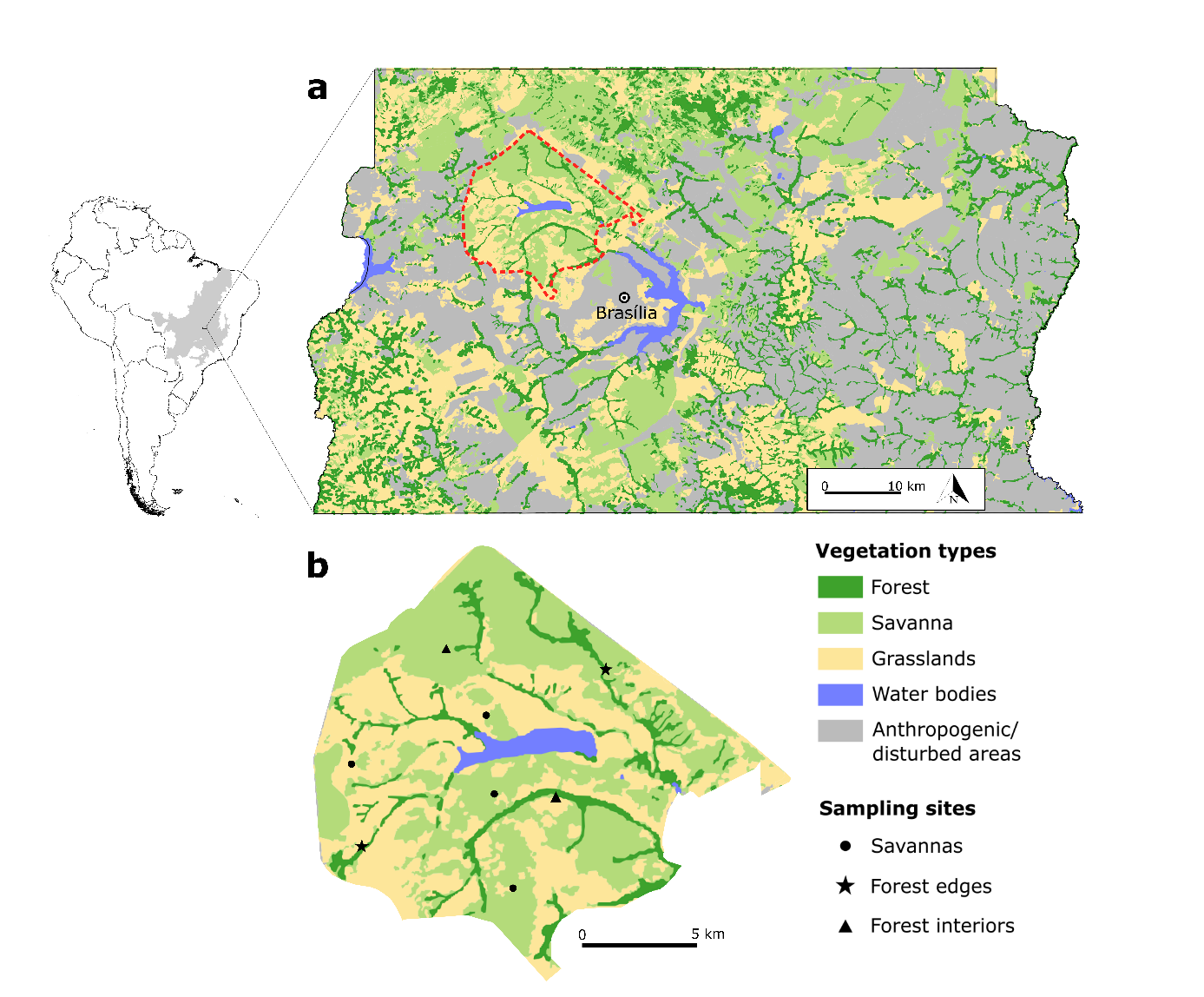
^

**Figure S1. Map illustrating the location and distribution of sampling sites in the Brasília National Park**. **a** - location of the Federal District within the Cerrado biome in South America (shaded area), where the Park (delimited by a dashed line) is located. Different colors on the map correspond to vegetation types. **b** – A close-up of the park, with sampling points marked according to the targeted vegetation type. Map created with QGIS 3.22.3 (<https://www.qgis.org/en/site/>). Land-use data from SISDIA – Federal District, Brazil (<https://www.metadados.sisdia.df.gov.br>).

**Table S1.** **Pollen morphotypes found on the body of bats throughout the twelve sampling months, identified to the lowest possible taxonomic level**. N - Frequency (and percentage in relation to the total number of interactions); n - the of sum of flowering individuals recorded in the transections; Habitat type - in which vegetation type they were reported (CSS – Cerrado *sensu stricto*, GFI – gallery forest interior, GFE – gallery forest edge), Chirop. - whether they are chiropterophilous or not; and floral shape. Dashes indicate that the species was found in pollen samples but not in site (n, habitat type), or that chiropterophily or floral shape may not be inferred by the taxonomical level achieved.

| Pollen type | N (%) | n | Habitat type | Chirop. | Floral shape | Label |
| --- | --- | --- | --- | --- | --- | --- |
| **Acanthaceae** |  |  |  |  |  |  |
| *Justicia* sp. | 1 (0.3) | - | - | No | - | Jusp |
| **Amaryllidaceae** |  |  |  |  |  |  |
| *Hippeastrum* *glaucescens* | 1 (0.3) | 1 | S | No | Infundibuliform | Hglau |
| **Arecaecae** |  |  |  |  |  |  |
| *Syagrus* type | 1 (0.3) | - | - | No | - | Asya |
| **Asteraceae** |  |  |  |  |  |  |
| *Baccharis* type | 1 (0.3) | - | - | No | - | Abacc |
| Vernoniae type | 1 (0.3) | - | - | - | - | Asver |
| **Caryocaraceae** |  |  |  |  |  |  |
| *Caryocar brasiliense* | 46 (15.2) | 95 | S | Yes | Brush | Cbra |
| **Combretaceae** |  |  |  |  |  |  |
| *Combretum fruticosum* | 5 (1.7) | 10 | I | No | Pseudanthium | Cfru |
| **Convolvulaceae** |  |  |  |  |  |  |
| *Ipomoeae procumbens* | 1 (0.3) | 1 | S | No | Infundibuliform | Ipro |
| *Merremia tomentosa* | 1 (0.3) | 4 | S | No | Infundibuliform | Mtom |
| **Cunoniaceae** |  |  |  |  |  |  |
| *Lamanonia ternata* | 23 (7.6) | 30 | E | No | Pseudanthium | Lter |
| **Fabaceae** |  |  |  |  |  |  |
| *Bauhinia* *goyazensis* group^1^ | 25 (8.3) | 46 | S/E | Yes | Tubular | Bgoy |
| *Bauhinia holophylla* | 11 (3.7) | 4 | S/E | Yes | Tubular | Bholo |
| *Bauhinia* *rufa* group^2^ | 30 (9.9) | 13 | E | Yes | Tubular | Brufa |
| Caesalpinoidae sp. 1 | 3 (1.0) | - | - | - | - | Fcae1 |
| Caesalpinoidae sp. 2 | 1 (0.3) | - | - | - | - | Fcae2 |
| Caesalpinoidae sp. 3 | 1 (0.3) | - | - | - | - | Fcae3 |
| *Hymenaea courbaril* | 10 (3.3) | 2 | I | Yes | Cupuliform | Hcou |
| *Hymenaea stigonocarpa* | 22 (7.3) | 6 | S | Yes | Cupuliform | Hsti |
| *Inga* aff. *laurina* | 4 (1.3) | - | - | Yes | Pseudanthium | Ilau |
| *Inga edulis* | 5 (1.7) | - | - | Yes | Tubular | Iedu |
| *Inga vera* | 8 (2.6) | - | - | Yes | Tubular | Iver |
| *Mimosa* aff. *setosa* | 1 (0.3) | 26 | S | No | Pseudanthium | Mset |
| **Loranthaceae** |  |  |  |  |  |  |
| *Psittacanthus robustus* | 35 (11.6) | 61 | S | No | Tubular | Prob |
| **Lyrthraceae** |  |  |  |  |  |  |
| *Lafoensia pacari* | 28 (9.3) | 2 | E | Yes | Campanulate | Lpac |
| **Malvaceae** |  |  |  |  |  |  |
| *Ceiba pentandra* | 13 (4.3) | - | I | Yes | Brush | Cpen |
| *Pseudobombax longiflorum* | 6 (2.0) | 21 | E | Yes | Brush/urceolate^3^ | Plon |
| *Pseudobombax tomentosum* | 4 (1.3) | 4 | S | Yes | Brush/urceolate^3^ | Ptom |
| **Moraceae** |  |  |  |  |  |  |
| Moraceae type | 2 (0.7) | - | - | - | - | Mora |
| **Myrtaceae** |  |  |  |  |  |  |
| *Eucalyptus* sp. | 3 (1.0) | - | - | Yes^4^ | Brush | Eusp |
| *Myrcia* sp. | 1 (0.3) | 31^5^ | S | No | Brush | Myrsp |
| Myrtaceae type | 1 (0.3) | - | - | - | - | Myrt |
| **Sapotaceae** |  |  |  |  |  |  |
| *Pouteria* type | 1 (0.3) | - | - | - | - | Spou |
| **Smilacaceae** |  |  |  |  |  |  |
| *Smilax* sp. | 1 (0.3) | - | - | No | Pseudanthium | Smil |
| **Undetermined** |  |  |  |  |  |  |
| Undetermined 1 | 1 (0.3) | - | - | - | - | Indet1 |
| Undetermined 2 | 1 (0.3) | - | - | - | - | Indet2 |

^1^ Includes the following species with similar flower and pollen morphology: *B. goyazensis* and *B. dumosa*;

^2^ Includes the following species with similar flower and pollen morphology: *B. rufa* and *B. longifoli;*

^3^ Flowers have an overall brush-like appearance, but the nectar chamber is formed by the large urceolate calyx;

^4^ Paleotropical chiropterophilous species introduced in the region for economic purposes and found around the PNB;

^5^  Abundance is the sum of *M. eryocalyx* and *M. multifloral*, the two species found in the site with similar floral morphologies.

**Table S2. Bat species captured during 12 sampling months in the Brasília National Park**. N - absolute number of captured individuals; rN - relative frequency; Habitat type – the vegetation type in which they were reported (S – savanna, I – gallery forest interior, E – gallery forest edge); pollen carriers - number (and percentage) of bats from each species that carried pollen; and the richness of plant species and/or pollen morphotypes found in their samples.

| Species | N | rN | Habitat type | | Pollen carriers (%) | | Pollen type richness | | Network label |  |  |
| --- | --- | --- | --- | --- | --- | --- | --- | --- | --- | --- | --- |
| **Molossidae** |  |  |  | |  | |  | |  |  |  |
| *Molossops temminckii* | 3 | 0.008 | E | | 0 (0.0) | | 0 | | - |  |  |
| **Mormoopidae** |  |  |  | |  | |  | |  |  |  |
| *Pteronotus rubiginosus* | 1 | 0.003 | I | | 0 (0.0) | | 0 | | - |  |  |
| **Phyllostomidae** |  |  |  | |  | |  | |  |  |  |
| Carollinae |  |  |  | |  | |  | |  |  |  |
| *Carollia perspicillata* | 62 | 0.161 | S/E/I | | 23 (37.1) | | 12 | | Cper |  |  |
| Desmodontinae |  |  |  | |  | |  | |  |  |  |
| *Diphylla ecaudata* | 1 | 0.003 | S | | 0 (0.0) | | 0 | | - |  |  |
| Glossophaginae |  |  |  | |  | |  | |  |  |  |
| *Anoura caudifer* | 24 | 0.062 | S/E/I | | 23 (95.8) | | 14 | | Acau |  |  |
| *Anoura geoffroyi* | 5 | 0.013 | S/E/I | | 5 (100.0) | | 8 | | Ageo |  |  |
| *Glossophaga soricina* | 43 | 0.111 | S/E/I | | 41 (95.3) | | 14 | | Gsor |  |  |
| Lonchophyllinae |  |  |  | |  | |  | |  |  |  |
| *Lonchophylla dekeyseri* | 22 | 0.057 | S/E | | 19 (86.4) | | 8 | | Ldek |  |  |
| Micronycterinae |  |  |  | |  | |  | |  |  |  |
| *Micronycteris schmidtorum* | 4 | 0.010 | S | | 3 (75.0) | | 3 | | Msch |  |  |
| Stenodermatinae |  |  |  | |  | |  | |  |  |  |
| *Artibeus concolor* | 1 | 0.003 | I | | 1 (100.0) | | 1 | | Acon |  |  |
| *Artibeus lituratus* | 79 | 0.205 | S/E/I | | 14 (17.7) | | 7 | | Alit |  |  |
| *Artibeus planirostris* | 24 | 0.062 | S/E/I | | 11 (45.8) | | 5 | | Apla |  |  |
| *Dermanura* cf*. anderseni* | 17 | 0.044 | S/E/I | | 1 (5.9) | | 2 | | Dand |  |  |
| *Dermanura cinerea* | 36 | 0.093 | S/E/I | | 5 (13.9) | | 4 | | Dcin |  |  |
| *Platyrrhinus lineatus* | 37 | 0.096 | S/E/I | | 14 (37.8) | | 9 | | Plin |  |  |
| *Sturnira lilium* | 13 | 0.034 | S/E/I | | 2 (15.4) | | 2 | | Slil |  |  |
| *Sturnira tildae* | 1 | 0.003 | E | | 0 (0.0) | | 0 | | - |  |  |
| **Vespertilionidae** |  |  |  | |  | |  | |  |  |  |
| *Eptesicus diminutus* | 1 | 0.003 | S | | 0 (0.0) | | 0 | | - |  |  |
| *Eptesicus furinalis* | 2 | 0.005 | S | | 0 (0.0) | | 0 | | - |  |  |
| *Lasiurus blossevillii* | 1 | 0.003 | E | | 0 (0.0) | | 0 | | - |  |  |
| *Myotis diminutus* | 1 | 0.003 | E | | 0 (0.0) | | 0 | | - |  |  |
| *Myotis riparius* | 2 | 0.005 | E/I | | 0 (0.0) | | 0 | | - |  |  |
| *Myotis nigricans* | 6 | 0.016 | E/I | | 0 (0.0) | | 0 | | - |  |  |
| **Total** | **386** |  | |  | |  | |  |  | |  |

**
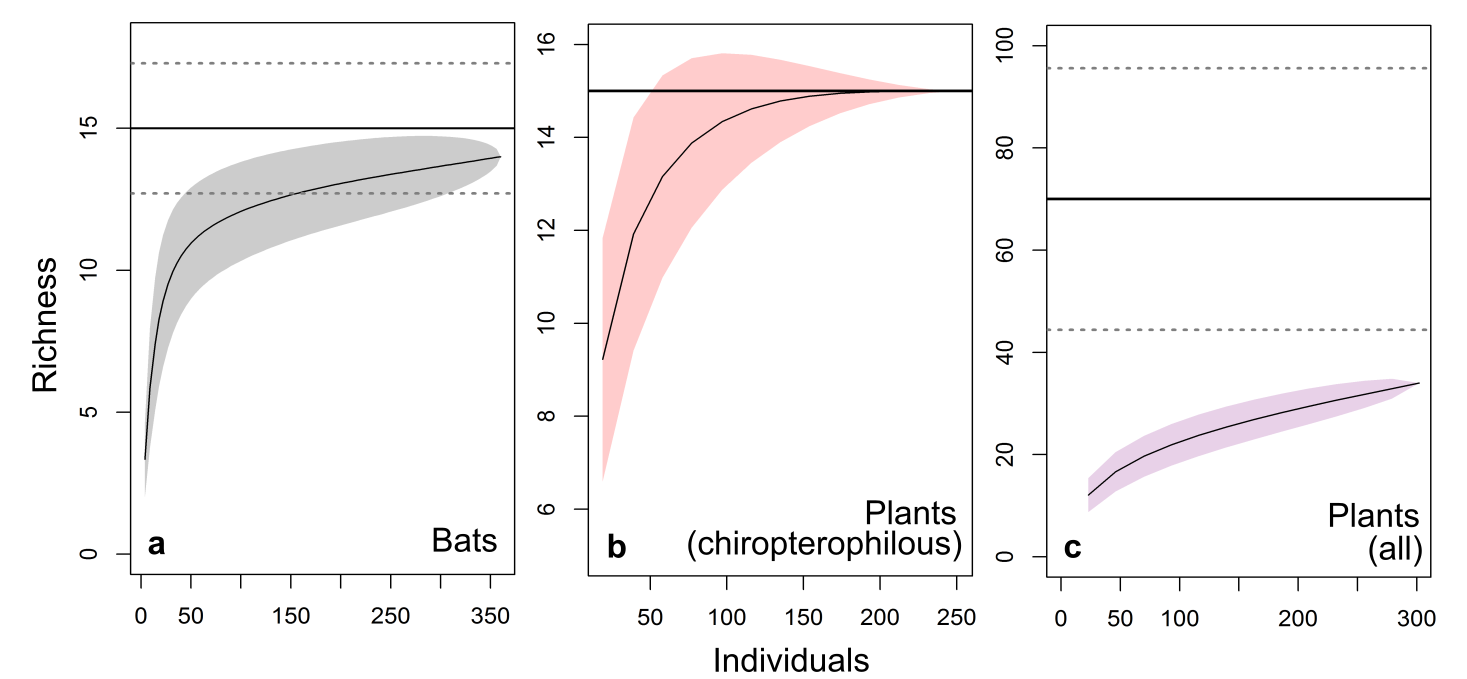
**

**Figure S2.** **Rarefaction curves.** a - bats species; b - pollen types from chiropterophilous plants; c - pollen types from all plants pooled. Shaded areas represent 95% confidence intervals, and solid and dashed horizontal lines indicate the estimated asymptotic richness and its standard error, respectively.

**Table S3. All plants registered in the transections that were classified as potential resources for bats, according to the criteria specified in the methods**. Habitat type: CSS – Cerrado *sensu stricto*, GFI – gallery forest interior, GFE – gallery forest edge), their pollination syndrome and floral shape.

| Espécie | Habitat type | Syndrome | Bat visitation | Floral shape |
| --- | --- | --- | --- | --- |
| **Acanthaceae** |  |  |  |  |
| *Justicia clivalis* | GFI | Ornithophily | No | Tubular |
| *Justicia irwinii* | GFI | Entomophily | No | Campanulate |
| *Ruellia incompta* | CSS | Entomophily | No | Infundibuliform |
| **Alstroemeriaceae** |  |  |  |  |
| *Alstroemeria gardneri* | CSS | Ornithophily | No | Infundibuliform |
| *Alstroemeria stenopetala* | GFE | Sphingophily | No | Campanulate |
| **Amaryllidaceae** |  |  |  |  |
| *Hippeastrum glaucescens* | CSS | Ornithophily | Yes | Infundibuliform |
| **Apocynaceae** |  |  |  |  |
| *Mandevilla illustris* | CSS | Entomophily | No | Infundibuliform |
| *Mandevilla pohliana* | CSS | Entomophily | No | Infundibuliform |
| *Odontadenia lutea* | CSS | Entomophily | No | Infundibuliform |
| **Arecaceae** |  |  |  |  |
| *Syagrus comosa* | CSS | Entomophily | No | Cupuliform |
| **Bignoniaceae** |  |  |  |  |
| *Adenocalymma*  *pedunculatum* | CSS | Entomophily | No | Infundibuliform |
| *Amphilophium elongatum* | GFE, GFI | Entomophily | No | Infundibuliform |
| *Anemopaegma acutifolium* | CSS | Entomophily | No | Infundibuliform |
| *Anemopaegma arvense* | CSS | Entomophily | No | Infundibuliform |
| *Arrabidaea sceptrum* | CSS | Entomophily | No | Infundibuliform |
| *Bignonia* cf. *corymbosa* | GFI | Entomophily | No | Infundibuliform |
| *Fridericia* cf. *florida* | GFI | Entomophily | No | Infundibuliform |
| *Fridericia platyphylla* | GFE | Entomophily | No | Infundibuliform |
| *Handroanthus chrysothrichus* | CSS | Entomophily | No | Infundibuliform |
| *Jacaranda caroba* | GFE | Entomophily | No | Infundibuliform |
| *Jacaranda ulei* | CSS | Entomophily | No | Infundibuliform |
| *Jacaranda* sp. | GFI | Entomophily | No | Infundibuliform |
| **Calophyllaceae** |  |  |  |  |
| *Kielmeyera abdita* | CSS | Entomophily | No | Rotate |
| *Kielmeyera coriaceae* | CSS, FGE | Entomophily | No | Rotate |
| *Kielmeyera pumila* | CSS | Entomophily | No | Rotate |
| *Kielmeyera variabilis* | CSS | Entomophily | No | Rotate |
| **Caryocaraceae** |  |  |  |  |
| *Caryocar brasiliense* | CSS | Chiropterophily | Yes | Brush |
| ***Celastraceae*** |  |  |  |  |
| *Plenckia populnea* | GFE | Entomophily | No | Pseudanthium |
| **Chrysobalanaceae** |  |  |  |  |
| *Couepia grandiflora* | CSS | Entomophily | No | Brush |
| **Combretaceae** |  |  |  |  |
| *Combretum fruticosum* | GFI | Ornithophily | Yes | Brush |
| **Convolvulaceae** |  |  |  |  |
| *Ipomoea sp.* | CSS | Entomophily | No | Infundibuliform |
| *Ipomoea procumbens* | CSS | Entomophily | Yes | Infundibuliform |
| *Jacquemontia velutina* | CSS | Entomophily | No | Infundibuliform |
| *Merremia digitata* var. *elongata* | CSS | Entomophily | No | Infundibuliform |
| *Merremia tomentosa* | CSS | Entomophily | Yes | Infundibuliform |
| **Cunoniaceae** |  |  |  |  |
| *Lamanonia ternata* | GFE | Entomophily | Yes | Pseudanthium |
| **Erythroxylaceae** |  |  |  |  |
| *Erythroxylum tortuosum* | CSS | Entomophily | No | Pseudanthium |
| **Fabaceae** |  |  |  |  |
| *Bauhinia dumosa* | GFE | Chiropterophily | Yes | Tubular |
| *Bauhinia goyazensis* | CSS, GFE | Chiropterophily | Yes | Tubular |
| *Bauhinia holophylla* | CSS | Chiropterophily | Yes | Tubular |
| *Bauhinia longifolia* | GFE | Chiropterophily | Yes | Tubular |
| *Bauhinia rufa* | GFE | Chiropterophily | Yes | Tubular |
| *Calliandra dysantha* | CSS, GFE | Ornithophily | No | Pseudanthium |
| *Hymenaea courbaril* | GFI | Chiropterophily | Yes | Cupuliform |
| *Hymenaea stigonocarpa* | CSS, GFE | Chiropterophily | Yes | Cupuliform |
| *Leptolobium dasycarpum* | GFE | Entomophily | No | Pseudanthium |
| *Mimosa clausseni* | CSS, GFE | Entomophily | No | Pseudanthium |
| *Mimosa regnelli* | CSS, GFE | Entomophily | No | Pseudanthium |
| *Mimosa setosa* | CSS, GFE | Entomophily | Yes | Pseudanthium |
| *Mimosa somnians* | CSS, GFE | Entomophily | No | Pseudanthium |
| *Stryphnodendron adstringens* | CSS | Entomophily | No | Pseudanthium |
| *Tachigali vulgaris* | CSS | Entomophily | No | Pseudanthium |
| **Gentianaceae** |  |  |  |  |
| *Calolisianthus speciosus* | CSS | Entomophily | No | Campanulate |
| *Sinningia elatior* | GFE | Ornithophily | No | Tubular |
| **Iridaceae** |  |  |  |  |
| *Gladiolus verdickii* | CSS | Entomophily | No | Cupuliform |
| **Lamiaceae** |  |  |  |  |
| *Hypenia macrantha* | CSS | Ornithophily | No | Tubular |
| *Amasonia hirta* | CSS | Entomophily | No | Tubular |
| *Rhabdocaulon denudatum* | GFE | Entomophily | No | Tubular |
| **Loranthaceae** |  |  |  |  |
| *Psittacanthus robustus* | CSS | Ornithophily | Yes | Tubular |
| **Lythraceae** |  |  |  |  |
| *Diplusodon* spp. | CSS, GFE | Entomophily | No | Rotate |
| *Diplusodon virgatus* | CSS, GFE | Entomophily | No | Rotate |
| *Lafoensia pacari* | GFE | Chiropterophily | Yes | Campanulate |
| **Malvaceae** |  |  |  |  |
| *Eriotheca pubescens* | CSS, GFE | Entomophily | No | Brush |
| *Luehea divaricata* | GFI | Entomophily | No | Brush |
| *Luehea grandiflora* | CSS, GFE | Chiropterophily | No | Cupuliform |
| *Pavonia rosa-campestris* | CSS, GFE | Entomophily | No | Rotate |
| *Peltaea polymorpha* | CSS, GFE | Entomophily | No | Rotate |
| *Pseudobombax longiflorum* | GFE | Chiropterophily | Yes | Brush/urceolate |
| *Pseudobombax tomentosum* | CSS | Chiropterophily | Yes | Brush/urceolate |
| **Myrtaceae** |  |  |  |  |
| *Campomanesia adamantium* | CSS | Entomophily | No(?) | Brush |
| *Campomanesia* cf. *aurea* | CSS | Entomophily | No(?) | Brush |
| *Campomanesia pubescens* | CSS | Entomophily | No(?) | Brush |
| *Campomanesia sessiliflora* | CSS | Entomophily | No(?) | Brush |
| *Myrcia eriocalyx* | CSS | Entomophily | Yes | Brush |
| *Myrcia* cf. *multiflora* | CSS, GFE | Entomophily | Yes | Brush |
| *Psidium sp.* | CSS | Entomophily |  | Brush |
| ***Orobanchaceae*** |  |  |  |  |
| *Esterhazya splendida* | CSS | Ornithophily | No | Tubular |
| **Protecaeae** |  |  |  |  |
| *Roupala montana* | CSS, GFE | Entomophily | No | Pseudanthium |
| **Rubiaceae** |  |  |  |  |
| *Ferdinandusa speciosa* | GFE | Ornithophily | No | Tubular |
| **Rutaceae** |  |  |  |  |
| *Spiranthera odoratissima* | CSS | Entomophily | No | Tubular |
| **Styracaceae** |  |  |  |  |
| *Styrax ferruginea* | CSS | Entomophily | No | Rotate |
| **Velloziaceae** |  |  |  |  |
| *Vellozia squamata* | CSS | Entomophily | No | Infundibuliform |
| **Vochysiaceae** |  |  |  |  |
| *Qualea grandiflora* | CSS | Entomophily | No | Calcarate |
| *Qualea multiflora* | CSS | Entomophily | No | Calcarate |
